# The microbiome of medicinal plants: diversity and importance for plant growth, quality and health

**DOI:** 10.3389/fmicb.2013.00400

**Published:** 2013-12-20

**Authors:** Martina Köberl, Ruth Schmidt, Elshahat M. Ramadan, Rudolf Bauer, Gabriele Berg

**Affiliations:** ^1^Institute for Environmental Biotechnology, Graz University of TechnologyGraz, Austria; ^2^Faculty of Agriculture, SEKEM, Heliopolis University, Ain Shams UniversityCairo, Egypt; ^3^Department of Pharmacognosy, Institute of Pharmaceutical Sciences, University of GrazGraz, Austria

**Keywords:** biocontrol, desert farming, medicinal plants, microbial communities, organic agriculture, soil-borne pathogens

## Abstract

Past medicinal plant research primarily focused on bioactive phytochemicals, however, the focus is currently shifting due to the recognition that a significant number of phytotherapeutic compounds are actually produced by associated microbes or through interaction with their host. Medicinal plants provide an enormous bioresource of potential use in modern medicine and agriculture, yet their microbiome is largely unknown. The objective of this review is (i) to introduce novel insights into the plant microbiome with a focus on medicinal plants, (ii) to provide details about plant- and microbe-derived ingredients of medicinal plants, and (iii) to discuss possibilities for plant growth promotion and plant protection for commercial cultivation of medicinal plants. In addition, we also present a case study performed both to analyse the microbiome of three medicinal plants (*Matricaria chamomilla* L., *Calendula officinalis* L., and *Solanum distichum* Schumach. and Thonn.) cultivated on organically managed Egyptian desert farm and to develop biological control strategies. The soil microbiome of the desert ecosystem was comprised of a high abundance of Gram-positive bacteria of prime importance for pathogen suppression under arid soil conditions. For all three plants, we observed a clearly plant-specific selection of the microbes as well as highly specific diazotrophic communities that overall identify plant species as important drivers in structural and functional diversity. Lastly, native *Bacillus* spec. div. strains were able to promote plant growth and elevate the plants’ flavonoid production. These results underline the numerous links between the plant-associated microbiome and the plant metabolome.

## THE PLANT-ASSOCIATED MICROBIOME: INTRODUCTION INTO STRUCTURE AND FUNCTION

All plant-associated microenvironments are colonized in high abundances by microorganisms, especially the nutrient-rich rhizosphere, the soil area influenced by plant roots, hosts a plethora of microbes that are of central importance for plant nutrition, health, and quality (Hiltner, 1904 in Hartmann et al., 2008; Berg, 2009; Mendes et al., 2011,^2013^). The rhizosphere can host up to 10^11^ microbial cells per gram root with more than 30,000 different prokaryotic species (Berendsen et al., 2012). These rhizospheric microorganisms from a highly diverse reservoir of soil microbes are attracted by the rhizosphere’s plant root secretions and other rhizodeposits (Compant et al., 2010), and driven via the compositional variability of these exudates (Bais et al., 2006; Doornbos et al., 2012). Each plant species harbors a specific rhizosphere microbiome dependent of the present soil community (Smalla et al., 2001). Besides plant species, the composition and diversity of microbial rhizosphere communities is shaped by soil type and pedoclimate, plant health and developmental stage, climate and season, pesticide treatments, grazers and animals, and several other biotic and abiotic factors (Singh and Mukerji, 2006; Berg and Smalla, 2009; Barnard et al., 2013). Some genera are ubiquitous and can be found distributed over the entire plant, such as the well-known plant-associated genera *Bacillus* and *Pseudomonas* (Berg et al., 2011). However, a high degree of specificity for each microenvironment was also observed via comparison of microbial colonization patterns of different microhabitats (Berg et al., 2005b; Fürnkranz et al., 2012; Köberl et al., 2013). Certain plant-associated microorganisms including beneficials and pathogens are also passed down from previous generations via the seed (Hardoim et al., 2012; Hirsch and Mauchline, 2012), and likewise a transmission between plants through pollen grains was recently observed (Fürnkranz et al., 2012). Interestingly, the phylogenetically oldest land plants, mosses, transfer a highly diverse core microbiome of primarily potential beneficial bacteria from the sporophyte to the gametophyte and *vice versa* (Bragina et al., 2012).

Medicinal plants harbor a distinctive microbiome due to their unique and structurally divergent bioactive secondary metabolites that are most likely responsible for the high specificity of the associated microorganisms (Qi et al., 2012). The analyses of several Chinese medicinal plant microbiomes showed interesting results (*Ainsliaea henryi* Diels, *Dioscorea opposita*, *Potentilla discolor* Bge, *Stellera chamaejasme* L., *Ophiopogon japonicus* (Thunb) Ker-Gawl., *Juncus effusus* L. var. *decipiens* Buchen., *Rhizoma arisaematis*, and others; Li et al., 2008; Zhao et al., 2011), as each of them hosted a specific actinobacterial community and showed a remarkably high and diverse rhizospheric and endophytic colonization with Actinobacteria featuring both antimicrobial and antitumor properties (Zhao et al., 2012). These Gram-positive and often spore-forming bacteria are promising biological control agents (BCAs), such as the genus *Streptomyces* that is a known and unique source of novel antibiotics (Goodfellow and Fiedler, 2010; Niraula et al., 2010; Nachtigall et al., 2011; Raaijmakers and Mazzola, 2012).

## MEDICINAL PLANTS: PLANT- AND MICROBE-DERIVED INGREDIENTS

Plants contain numerous different biologically active compounds, and plant-derived medicines have been part of traditional healthcare in most parts of the world for thousands of years. Traditional Chinese medicine (TCM), phytotherapeutic knowledge from the Mayans, the aboriginal medicine of Australia, and several other cultures comprise a huge spectrum of natural remedies that can be exploited as sources for new and effective therapeutic agents. Still widely practiced in the modern era, TCM supplies ethnopharmaceutical knowledge on over 5,000 plant species used for the treatment of numerous diseases and has already provided the basis for the discovery of many modern drugs, such as anticancer agents (Miller et al., 2012a,2012b). In general, natural products play a highly considerable role in the drug discovery and development process, as about 26% of the new chemical entities introduced into the market worldwide from 1981 to 2010 were either natural products or those derived directly therefrom, reaching a high of 50% in 2010 (Newman and Cragg, 2012). In the past, medicinal plant research focused primarily on their ingredients, however, recently the focus has shifted to include the structure and function of several medicinal plant microbiomes. Surprisingly, not only were the plants themselves able to produce substances with phytotherapeutic properties, but their associated microbes, in particular endophytes, could as well (**Table 1**). Currently, research continues to show that a significant number of natural products are actually produced by microbes and/or microbial interactions with the host from whence they were isolated (Gunatilaka, 2006), and for several medicinal plants it is presumed that the plant-associated microbiome, especially the complex community of the endomicrobiome, is directly or indirectly involved in the production of bioactive phytochemicals. Presently, however, only a small subset of potential microbial strains could be definitively attributed to phytotherapeutic properties (Strobel and Daisy, 2003; Strobel et al., 2004; Chandra, 2012; Miller et al., 2012a,b), and their relative contribution to the recognized valuable bioactivity of medicinal plants is not clear as of yet.

**Table 1:** Examples for bioactive phytometabolites where microorganisms are involved in their production.

Bioactive compound	Therapeutic properties	Host plant	Producing microorganism	Reference
Munumbicins	Antibacterial, antimycotic, antiplasmodial	*Kennedia nigriscans*	*Streptomyces* sp.**	Castillo etal. (2002)
Kakadumycins	Antibacterial, antiplasmodial	*Grevillea pteridifolia*	*Streptomyces* sp.**	Castillo etal. (2003)
Coronamycins	Antimycotic, antiplasmodial	*Monstera* sp.**	*Streptomyces* sp.**	Ezra etal. (2004)
Oocydin A	Antimycotic (Oomycota)	*Rhyncholacis penicillata*	*Serratia marcescens*	Strobel etal. (1999a)
Cryptocandin	Antimycotic	*Tripterigeum wilfordii*	*Cryptosporiopsis quercina*	Strobel etal. (1999b)
Colletotric acid	Antibacterial, antimycotic	*Artemisia mongolica*	*Colletotrichum gloeosporioides*	Zou etal. (2000)
Artemisinin	Antiplasmodial	*Artemisia annua*	*Colletotrichum* sp.**	Wang etal. (2001)
Cochliodinol	Antibacterial, antimycotic, anticancer	*Salvia officinalis*	*Chaetomium* sp.**	Debbab etal. (2009)
Botryorhodines	Antimycotic, anticancer	*Bidens pilosa*	*Botryosphaeria rhodina*	Abdou etal. (2010)
Pestacin and Isopestacin	Antimycotic, antioxidant	*Terminalia morobensis*	*Pestalotiopsis microspora*	Strobel etal. (2002), Harper etal. (2003)
Phomol	Antiphlogistic, antibacterial, antimycotic, anticancer	*Erythrina crista-galli*	*Phomopsis* sp.**	Weber etal. (2004)
Podophyllotoxin	Anticancer, antiphlogistic	*Podophyllum hexandrum; Juniperus communis*	*Alternaria* sp.; *Aspergillus fumigatus*	Yang etal. (2003), Kusari etal. (2009a)
Paclitaxel (Taxol)	Anticancer	*Taxus brevifolia; Ginkgo biloba; Aloe vera*	*Taxomyces andreanae; Alternaria* sp.; *Phoma* sp.	Wani etal. (1971), Stierle etal. (1993), Kim etal. (1999), Immaculate etal. (2011)
Camptothecin	Anticancer, antiviral (HIV)	*Nothapodytes foetida; Camptotheca acuminate*	*Entrophospora infrequens; Fusarium solani*	Puri etal. (2005), Amna etal. (2006), Kusari etal. (2009b)
Maytansine	Anticancer	*Putterlickia verrucosa*	*Actinosynnema pretiosum*	Wings etal. (2013)
Rohitukine	Antiphlogistic, anticancer, immunomodulatory	*Dysoxylum binectariferum*	*Fusarium proliferatum*	Mohana Kumara etal. (2012)
Subglutinols	Immunomodulatory	*Tripterigeum wilfordii*	*Fusarium subglutinans*	Lee etal. (1995)

In regards to the alarming incidence of antibiotic resistance in bacteria with medical relevancy, medicinal plants with antibacterial properties are of central importance as bioresources for novel active metabolites (Palombo and Semple, 2001). Likewise, there is an increasing need for more and better antimycotics to treat those with weakened immune systems who are more prone to developing fungal infections, such as from the AIDS epidemic, cancer therapy, or organ transplants (Strobel and Daisy, 2003; Strobel et al., 2004). For centuries, several phytotherapeutics have also been known for their antiphlogistic features, yet despite the progress within medical research, chronic inflammatory diseases such as asthma, arthritis, and rheumatism remain one of the world’s leading health problems (Li et al., 2003). Hypertension is another critical issue for human health and is a primary risk factor for stroke, heart disease, and renal failure. Many herbal remedies as well as foods, however, are known and effective folk medicines in the prevention and/or treatment of high blood pressure (Abdel-Aziz et al., 2011). Hence, nature must still harbor plenty of currently unknown active agents that may serve as leads and scaffolds for the development of desperately needed efficacious drugs for a multitude of diseases (Newman and Cragg, 2012). Today, globalization has also had an impact on the use of medicinal plants and has proven beneficial in allowing greater access to these medicines for people all across the globe. For example, TCM plants are very popular in Europe, whereas the traditional German chamomile is primarily produced in Egypt. Growth, quality, and health of the medicinal plants are highly influenced and controlled by their microbiota through microbial metabolisms and host interactions.

## PLANT GROWTH PROMOTION AND BIOLOGICAL CONTROL FOR MEDICINAL PLANTS

Several rhizospheric microbes interact beneficially via different mechanisms with their host plant. They can have a direct plant growth promoting effect based on improved nutrient acquisition or hormonal stimulation, or indirectly affect the plant health by suppression of phytopathogens (Berg, 2009; Lugtenberg and Kamilova, 2009). Biofertilizers are microbes that supply the plant with nutrients, for example symbiotic root-nodulating rhizobia are the most prominent among the nitrogen-fixing microorganisms. Other microbial biofertilizers, such as mycorrhizal fungi and several rhizobacteria, are able to solubilise plant-available phosphate from either organic or inorganic bound phosphate (Lugtenberg et al., 2002). Microbes that hormonally promote plant growth are termed phytostimulators, and the phytohormone auxin, for instance, produced by fluorescent pseudomonads is one of the best understood examples (Kamilova et al., 2006; Khare and Arora, 2010). Various rhizobacteria, including for example *Burkholderia cepacia*, *Staphylococcus epidermidis,* and strains of the *Bacillus subtilis* group, stimulate plant growth by the emission of volatile organic compounds (VOCs; Ryu et al., 2003; Kai et al., 2007; Effmert et al., 2012; Bitas et al., 2013). VOCs are low molecular weight molecules (<300 Da) that have high vapor pressures and are therefore able to diffuse over long distances through the porous structure of the soil and through water-filled pores (Kai et al., 2007; Insam and Seewald, 2010). Indirectly, the plant growth can be promoted via biological control of phytopathogens. Pathogen growth can be inhibited by antibiotics or VOCs, toxins, biosurfactants, or extracellular cell wall-degrading enzymes, but microbial antagonism can also occur via degradation of pathogenicity factors like toxins, or simply by the competition for nutrients, minerals, or colonization sites (Berg, 2009). Another possible way to reduce the activity of pathogenic microorganisms is the activation of the plant defense mechanisms, or the so called induced systemic resistance (ISR) triggered by certain non-pathogenic rhizobacteria. Flagella, lipopolysaccharides, siderophores, VOCs, and several other bacterial components are thought to be involved in activating the non-pathogenic rhizobacteria-mediated ISR signaling pathway (van Loon et al., 1998; Lugtenberg and Kamilova, 2009).

Biological control of plant pathogens as well as plant growth promotion with microorganisms has been intensively studied over the past decades and is becoming a realistic alternative to chemical pesticides and fertilizers in sustainable agriculture (Weller, 2007). Several microbial inoculants have already been successfully commercialized (Berg, 2009, 2013), but a specific biological control strategy for medicinal plants, which are increasingly affected by different soil-borne phytopathogens, has not been available until now. While specific biocontrol agents for medicinal plants are needed, their associated microbiomes with outstanding metabolic activities also provide a promising source for novel BCAs.

## MEDICINAL PLANTS AND (POTENTIAL) HUMAN PATHOGENS: OCCURRENCE AND POSSIBLE BIOCONTROL

Traditional medicinal plants are often consumed raw, such as berries or other edible fruits, or in dried form as herbal brews or teas. Therefore, it is of crucial importance that any potentially harmful effect of associated microorganisms or of an applied biocontrol agent on human health be avoided completely. Recently, for instance, bacterial strains closely related to *Stenotrophomonas maltophilia* and *Rhodococcus* sp. were isolated from the roots of oregano (*Origanum vulgare* L.) cultivated in a sub-Himalayan region (Bafana, 2013). Similarly, *Ochrobactrum* and *Rhodococcus* were also detected on the studied medicinal plants in Egypt (*Matricaria chamomilla* L., *Calendula officinalis* L., and *Solanum distichum* Schumach. and Thonn.; Köberl et al., 2011). Among several others, these bacterial genera are known for their ambiguous interactions with eukaryotic hosts whereby the mechanisms responsible for plant growth promotion are similar to those also responsible for opportunistic infections in humans and animals (Berg et al., 2005a). In addition to the suppression of phytopathogens, antagonistic activity against potentially harmful human pathogens should also be considered in the biocontrol strategy.

Conversely, ethanolic extracts from the Chinese medicinal plants *Mallotus yunnanensis* Pax et. Hoffm., *Schima sinensis* (Hemsl. et. Wils) Airy-Shaw., *Garcinia morella* Desr., *Evodia daneillii* (Benn) Hemsl., *Meliosma squamulata* Hance., *Skimmia arborescens* Anders., and *Brandisia hancei* Hook. f. were determined as highly active against the clinical pathogens *Staphylococcus aureus*, *Escherichia coli*, *Pseudomonas aeruginosa,* and *Candida albicans* which corresponds to their traditional applications in skin and other infections (Zuo et al., 2012). Promising antimicrobial activities against human multi-drug-resistant pathogens have been observed for Mexican medicinal plants as well (Jacobo-Salcedo Mdel et al., 2011). As previously discussed for phytotherapeutic properties, the suppression of human pathogens can also be frequently attributed to medicinal plant-associated microbes and their secondary metabolites (Miller et al., 2012b; Mousa and Raizada, 2013).

In conclusion, medicinal plants should be considered as meta-organisms that comprise both the plant themselves and their microbiome. As meta-organisms, they are a largely untapped and enormous bioresource for bioactive compounds and microorganisms of potential use in modern medicine, agriculture, and pharmaceutical industry. As such, more research is necessary to exploit this immense reservoir for mankind.

## A CASE STUDY: THE MICROBIOME OF MEDICINAL PLANTS GROWN ON A DESERT FARM UNDER ORGANIC MANAGEMENT

**FIGURE 1:**
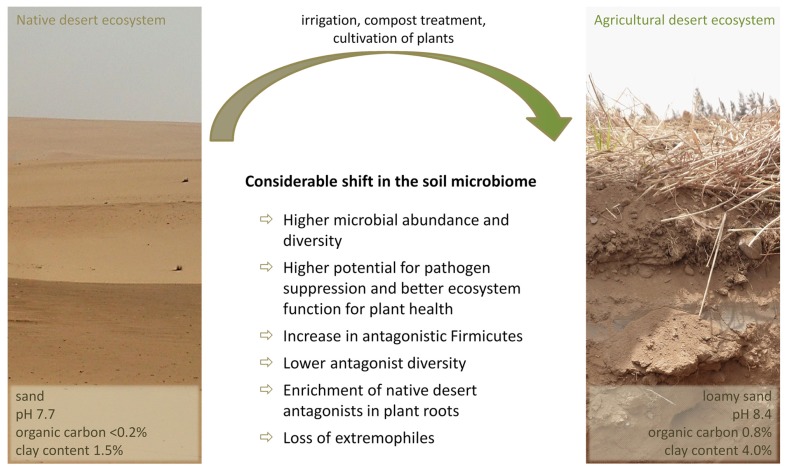
**Community shift in the soil microbiome after long-term agricultural use of the desert under organic management**.

In comparison to soils of humid areas, the soil microbiome of the Egyptian desert farm Sekem was comprised of a high abundance of Gram-positive, spore-forming bacteria primarily of the Firmicutes branch with 37% of the total bacterial soil community as revealed through a pyrosequencing-based amplicon sequencing approach (Köberl et al., 2011). However, a global soil community analysis including 32 libraries of 16S rRNA and 16S rRNA gene libraries from a variety of soils reported Firmicutes contribute a mean of only 2% in the total bacterial soil community (Janssen, 2006). *Bacillus* and *Paenibacillus* play the key role in explaining this remarkably high abundance of Firmicutes in the investigated desert agro-ecosystem. These drought-resistant genera are of prime importance for pathogen suppression under arid conditions as nearly all isolated antagonists with activity against soil-borne phytopathogenic fungi could be affiliated to this taxonomic group. This is in direct contrast to humid soils, where primarily Gram-negative bacteria like *Pseudomonas* are responsible for the indigenous antagonistic potential (Berg et al., 2005b; Haas and Défago, 2005; Costa et al., 2006; Zachow et al., 2008). A significantly higher proportion of Firmicutes and antifungal isolates were observed in field soil from the Egyptian farm than in the surrounding desert soil uninfluenced by human activities. In general, the total bacterial soil microbiome of the anthropogenic ecosystem exhibited a higher diversity and better ecosystem function for plant health in comparison to the natural desert soil (**Figure 1**). Due to the long-term agricultural use of the desert and the associated increasing occurrence of plant pathogens, the indigenous antagonistic potential in soil was almost twice as high as in the uncultivated desert soil. However, the diversity of antagonistic bacteria was lower and highly dominated by isolates of the *Bacillus subtilis* group. The most efficient antagonists from the native desert soil belonged to *Streptomyces*, and *Bacillus* and *Paenibacillus* species were the most frequently isolated antagonists from all investigated arid habitats including both desert and agriculturally used soil, as well as from the rhizosphere and endorhiza of three different species of medicinal plants cultivated on the desert farm (*Matricaria chamomilla* L., *Calendula officinalis* L., and *Solanum distichum* Schumach. and Thonn.). None of the plants are native to Egypt, and therefore were exposed to a previously unencountered microbiome. Interestingly, despite a clearly plant-specific selection of the associated bacterial microbiome, indigenous *Bacillus* and *Paenibacillus* strains of native desert soil with promising antagonistic properties against a wide range of soil-borne phytopathogens were enriched in all investigated plant roots. Conversely, several extremophilic bacterial groups, such as *Acidimicrobium*, *Rubellimicrobium*, and *Deinococcus*–*Thermus* decreased or completely disappeared from soil after agricultural use (Köberl et al., 2011).

**FIGURE 2:**
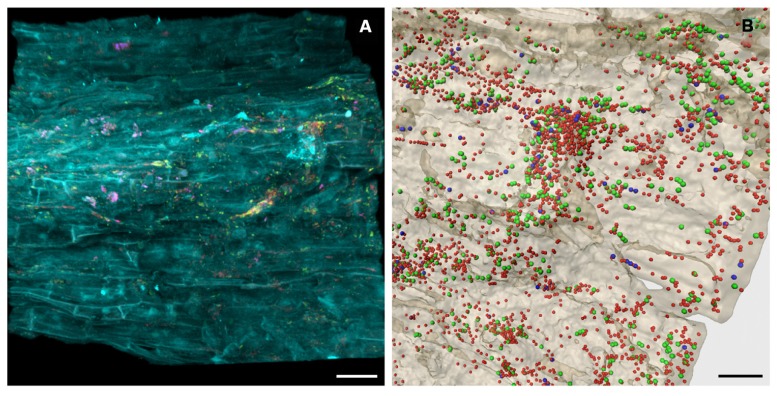
***In situ* visualization of the bacterial root colonization of *Matricaria chamomilla*.** Volume rendering **(A)** and three-dimensional reconstruction model **(B)** of confocal laser scanning microscopy stacks. **(A)** yellow = Alphaproteobacteria, pink = Betaproteobacteria, red = other eubacteria, cyan = root tissue, scale bar = 30 μm. **(B)** green = Alphaproteobacteria, blue = Betaproteobacteria, red = other eubacteria, beige = root tissue, scale bar = 15 μm.

Nitrogen is an essential macronutrient for plants and one of the most yield-limiting factors in agricultural production systems throughout the world (Bhattacharjee et al., 2008; Orr et al., 2011). To gain insight into the indigenous community of diazotrophic plant growth promoting microorganisms that inhabit desert agro-ecosystems, community profiles of the *nifH* gene encoding the nitrogenase reductase subunit were assessed. A broad diversity and high abundance of diazotrophs were detected in all investigated habitats, thus underlining their importance in native and agricultural desert ecosystems. Due to watering and cultivation of desert soil, a considerable shift toward a higher abundance and diversity was also observed for the nitrogen-fixing community. Phylogenetic analyses distinguishing between the major *nifH* gene types (Zehr et al., 2003; Gaby and Buckley, 2012) revealed that all NifH sequences from soil libraries were affiliated with the canonical *nifH* clusters I (conventional molybdenum nitrogenases) and III (molybdenum nitrogenases from anaerobes), while no sequences of alternative nitrogenases (cluster II) and *nifH* paralogs (clusters IV and V) were found. In general, the diazotrophic soil microbiota was highly dominated by NifH sequences related to Alphaproteobacteria. Each investigated medicinal plant cultivated on the desert farm harbored a specific root-associated diazotrophic microbiome. The rhizosphere inhabitants of *Matricaria chamomilla* (**Figure 2**) and *Calendula officinalis* were similar and both dominated by potential root-nodulating rhizobia acquired mainly from soil. Conversely, the rhizosphere of *Solanum distichum* was colonized in higher abundances by free-living nitrogen fixers most likely transmitted between plants as they were undetectable in soils. Although well-known for taxonomic community structure (Berg and Smalla, 2009; Bulgarelli et al., 2012), this high degree of plant-specificity identified plants as important drivers for functional diversity as well (Köberl, 2013). The total bacterial and fungal communities also revealed similar colonization patterns between the medicinal plants *Matricaria chamomilla* and *Calendula* officinalis compared to *Solanum distichum* (Köberl et al., 2013). This effect may have been intensified as a result of the close relationship between *Matricaria chamomilla* and *Calendula officinalis* who both belong to the Asteraceae family and therefore produce more similar bioactive metabolites. Furthermore, both *Matricaria chamomilla* and *Calendula officinalis* are annual herbal medicinal plants, while *Solanum distichum* is a perennial plant thus providing a longer timeframe to specifically select a stable associated microbiome.

In contrast to the highly specific bacterial communities associated with cultivated medicinal plants, fungal communities were less discriminative and characterized primarily by potential pathogens. Phytopathogenic species *Fusarium*, *Verticillium*, and several others were frequently identified, and, apart from *Rhizoctonia*, were the main soil-borne pathogens on the investigated desert farm that caused high yield losses on a wide host range of economically important crops, including the medicinal plants. To biologically control these soil-borne diseases, different desert habitats were screened for potential BCAs adapted to the unique and arid conditions of desert farming. Due to this high content of potential plant pathogens in the fungal community, the selection of antagonists was focused on the indigenous bacterial microbiome. An *in vitro* screening of 1,212 bacterial isolates linked with the comprehensive ecological data resulted in an antagonist collection of 45 genotypically different antifungal strains. In a hierarchical evaluation including their antifungal properties against *Verticillium dahliae*, *Rhizoctonia solani,* and *Fusarium culmorum* in addition to their antagonistic activity against the soil-borne plant pathogenic bacterium *Ralstonia solanacearum* and the nematode *Meloidogyne incognita*, three promising drought- and heat-resistant biocontrol candidates were selected: *Streptomyces subrutilus* Wb2n-11 isolated from desert soil in Sinai, *Bacillus subtilis* subsp. *subtilis* Co1-6 obtained from the rhizosphere of *Calendula officinalis*, and *Paenibacillus polymyxa* Mc5Re-14 isolated from the endorhiza of *Matricaria chamomilla*. Each belongs to risk group 1 and poses no risk for humans or the environment. These three potential BCAs have already shown promising *in vitro* plant growth promoting activities and stress tolerances; *Bacillus subtilis* Co1-6 exhibited high drought and salt resistance, protease and glucanase activity, and the production of siderophores, *Paenibacillus polymyxa* Mc5Re-14 had a lower tolerance to abiotic stresses in comparison to the *Bacillus* strain, but also tested positive for siderophores and glucanase activity, and the desert bacterium *Streptomyces subrutilus* Wb2n-11 showed hydrolytic degradation of chitin and glucan. All of them produced antibiotics against the nematode *Meloidogyne incognita*, however, their antibacterial activities were highly specific. While *Bacillus subtilis* and *Streptomyces subrutilus* exhibited antagonistic suppression of the plant pathogen *Ralstonia solanacearum*, only the *Paenibacillus* isolate was active against the opportunistic human pathogen *Escherichia coli* (Köberl et al., 2013).

These three autochthonous Gram-positive strains were selected for *ad planta* evaluation in the field under desert farming conditions in comparison to three allochthonous Gram-negative strains already known for their beneficial plant–microbe interactions in humid soils: *Pseudomonas fluorescens* L13-6-12 isolated from the rhizosphere of potato (*Solanum tuberosum*), *Stenotrophomonas rhizophila* P69 from oilseed rape (*Brassica napus*) rhizosphere, and *Serratia plymuthica* 3Re4-18 from the endorhiza of potato (Lottmann and Berg, 2001; Wolf et al., 2002; Kai et al., 2007; Zachow et al., 2010; Alavi et al., 2013). The first results revealed that priming chamomile seedlings with the autochthonous strains not only showed a stabilizing effect on plant performance, but *Bacillus subtilis* Co1-6 and *Paenibacillus polymyxa* Mc5Re-14 were also able to further elevate the plants’ flavonoid production. Higher contents of the bioactive compounds apigenin-7-*O*-glucoside and apigenin, which belong to the major flavonoids of chamomile florets (Kato et al., 2008; Srivastava and Gupta, 2009), were measured in blossoms of plants treated with the two Bacillales strains compared to blossoms of other treatments and uninoculated control plants (Schmidt et al., 2013). These findings demonstrate that a targeted bacterial treatment could influence the metabolic activity of the plant, and therefore represent one of the many poorly understood links between the structure and metabolic profile of the plant-associated microbiome and the plant metabolome.

## AUTHOR CONTRIBUTIONS

Conceived and designed the experiments: Gabriele Berg, Rudolf Bauer, Elshahat M. Ramadan and Martina Köberl. Performed the experiments: Martina Köberl and Ruth Schmidt. Analyzed the data: Martina Köberl, Ruth Schmidt and Gabriele Berg. Contributed reagents/materials/analysis tools: Gabriele Berg. Wrote the paper: Martina Köberl and Gabriele Berg.

## Conflict of Interest Statement

The authors declare that the research was conducted in the absence of any commercial or financial relationships that could be construed as a potential conflict of interest.
